# Bath Chalyeate Spring

**Published:** 1829

**Authors:** 


46. *Bath Chalybeate Spring.*—In the 3d No. of our 2d vol. we gave an extended account of several mineral springs in Kentucky and Ohio. Many others are known to exist in the western states; and, doubtless, but a part of them have yet been discovered. Regarding it as important for our practitioners of medicine, to be informed of the location and composition of these *fountains of health*, or, at least, *waters of hope and cheerfulness*, we shall, from time to time, announce those of which we can speak from personal observation, or from information on which we can rely. At present we shall confine ourselves to a brief notice of a chalybeate spring, which has received the name prefixed to this article. It is situated in Union county Indiana, about 8 miles from Brookville, and 44 from Cincinnati.

We have been furnished with a specimen of its waters, and have ascertained, that they are impregnated with the proto-carbonate of iron, and are identical, in composition, with the waters of the 'yellow spring,' in Ohio, and the chalybeate vein, at the Olympian springs, in Kentucky; of both of which, we have given some account in the paper already mentioned.

From Dr. Dunlavy, a respectable physician of Hamilton in this state, we have received a short topographical and geological account of its situation. The scenery is pleasant and romantic; and the spot itself with the surrounding country healthy. The spring bursts out from a high alluvial bank, and in its course deposits a sediment of per-oxide and carbonate of iron, like the 'yellow spring;' while the calcareous rocks beneath, are of the same variety with those observed at the latter fountain.

On the whole we may recommend this new watering place, to all who may find its location convenient; and are authorized to assure them, that comfortable accommodations have been already made for their reception.

